# DNA fragility at the *KMT2A*/*MLL* locus: insights from old and new technologies

**DOI:** 10.1098/rsob.220232

**Published:** 2023-01-11

**Authors:** Ian G. Cowell, Caroline A. Austin

**Affiliations:** Biosciences Institute, The Faculty of Medical Sciences, Newcastle University, Newcastle upon Tyne NE2 4HH, UK

**Keywords:** MLL, KMT2A, chromosome translocation, DNA topoisomerase II, etoposide, leukaemia

## Abstract

The *Mixed-Lineage Leukaemia* (*MLL/KMT2A*) gene is frequently rearranged in childhood and adult acute leukaemia (AL) and in secondary leukaemias occurring after therapy with DNA topoisomerase targeting anti-cancer agents such as etoposide (t-AL). *MLL/KMT2A* chromosome translocation break sites in AL patients fall within an 8 kb breakpoint cluster region (BCR). Furthermore, *MLL/KMT2A* break sites in t-AL frequently occur in a much smaller region, or hotspot, towards the 3′ end of the BCR, close to the intron 11/exon 12 boundary. These findings have prompted considerable effort to uncover mechanisms behind the apparent fragility of the BCR and particularly the t-AL hotspot. Recent genome-wide analyses have demonstrated etoposide-induced DNA cleavage within the BCR, and it is tempting to conclude that this cleavage explains the distribution of translocation break sites in t-AL. However, the t-AL hotspot and the centre of the observed preferential DNA cleavage are offset by over 250 nucleotides, suggesting additional factors contribute to the distribution of t-AL break sites. We review these recent genomic datasets along with older experimental results, analysis of TOP2 DNA cleavage site preferences and DNA secondary structure features that may lead to break site selection in t-AL *MLL/KMT2A* translocations.

## Graphical abstract

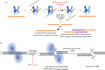

## 1. Background

**Figure 1.**
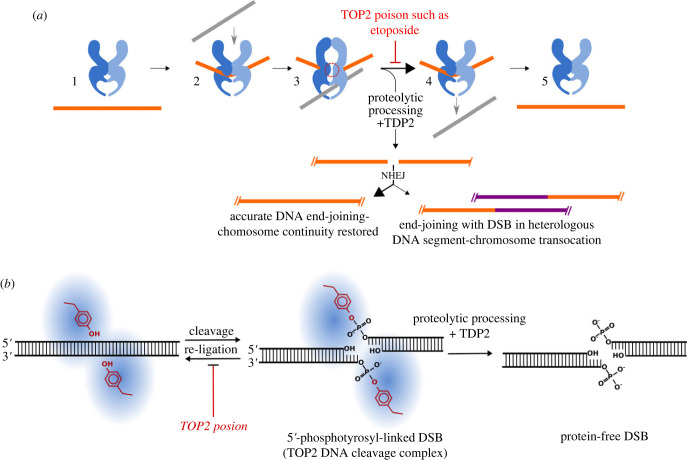
TOP2 strand passage activity and chromosomal translocation mechanism. (*a*) The TOP2 dimer binds to one DNA duplex (G segment, orange, 1–2) and cleaves both strands in a staggered cut with a 4-bp 5′overhang through which the enzyme protomers remain attached via a 5′-phosphotyrosyl linkage. A second DNA duplex (T segment, grey) passes through the transient enzyme-coupled break (highlighted by a red dashed circle, 2–3) driven by ATP-dependent enzyme conformational changes. The first duplex (G segment) is then re-ligated, and the products of the reaction are released from the enzyme (4–5). TOP2 poisons such as etoposide block the re-ligation step resulting in the accumulation of stabilized TOP2-DNA complexes know as cleavage complexes (CCs) which can be converted to protein-free DSBs with a 4 bp overhang by proteasomal action and TDP2. Repair of these breaks leads to the potential for chromosome translocation. (*b*) Molecular detail of TOP2 DNA cleavage. TOP2 generates a staggered break via nucleophilic attack by the active site tyrosine on the DNA sugar phosphate backbone of the G-segment. The resulting covalent TOP2-5′phosphotyrosyl–DNA complexes are normally transient and reversible but are stabilized by TOP2 poisons. Cellular processing generates protein free-ligatable breaks that are the substrate for NHEJ.

TOP2 poisons such as etoposide are anti-cancer agents that are widely employed to treat solid tumours and haematological malignancies. These cytotoxic drugs are effective, but their use is associated with significant side effects including increased risk of therapy-related acute leukaemias (t-AL), especially acute myeloid leukaemias (t-AML) that often harbour characteristic balanced chromosome translocations [1]. The most commonly involved locus in these translocations is *Mixed Lineage Leukaemia* (*MLL*), otherwise known as *Lysine methyl transferase 2A* (*KMT2A*) at 11q23. *MLL/KMT2A* chromosome translocations have been reported in acute leukaemia with over one hundred partner genes [2–4], most frequently *AF4* (*MLLT2*), *AF9* (*MLLT3*) and *ENL* (*MLLT1*). Other recurrent chromosome translocations encountered in t-AL include t(15;17)(*PML-RARA*), t(8;21)(*AML/RUNX1-ETO*) and inv(16)(*MYH11-CBFB*) [3]. These chromosome translocations are crucial early events in the development of the leukaemias and the resulting fusion genes, for example, *MLL-AF9*, are able to transform haematopoietic precursors and induce leukaemia in animal models [5,6]. TOP2 poisons block the completion of the reaction cycle of DNA topoisomerase II (TOP2). TOP2 enzymes (TOP2A and TOP2B in vertebrates) regulate DNA supercoiling and catenation by allowing one double-stranded DNA segment to pass through another via a normally short-lived TOP2-bridged double-strand break (DSB) in the first duplex (figure 1*a*). In this transient state each protomer of the dimeric enzyme is covalently attached to the ends of the DSB via a 5′-phosphotyrosyl linkage (figure 1*b*). A second DNA segment is then passed through the enzyme-bridged DNA gate, and the break is ultimately re-ligated with no change in DNA sequence. TOP2 poisons inhibit this re-ligation step, resulting in the formation of an unusual type of DSB called a cleavage complex (CC), in which the topoisomerase protein remains covalently linked to the DNA and the DNA break remains buried in the protein-DNA complex. These TOP2-linked breaks are cytotoxic, hence the utility of TOP2 poisons in cancer therapy, and they can be processed in the cell to reveal protein-free DSBs (figure 1*b*), which then elicit a DNA damage response [7–12]. This processing is a necessary step in repair of TOP2 CCs [13,14] which subsequently occurs largely via non-homologous end-joining (NHEJ) [15–17]. While this repair is typically error free, etoposide-linked TOP2-mediated DNA cleavage may directly contribute to oncogenic chromosome translocation events via misdirected joining of different chromosomal segments by NHEJ (figure 1*a*). The erroneous joining of DNA ends from different chromosome segments presumably requires close juxtaposition of these segments, and we have previously proposed that this may be facilitated by ongoing transcription in shared RNA polymerase clusters/transcription factories [18,19].

**Figure 2.**
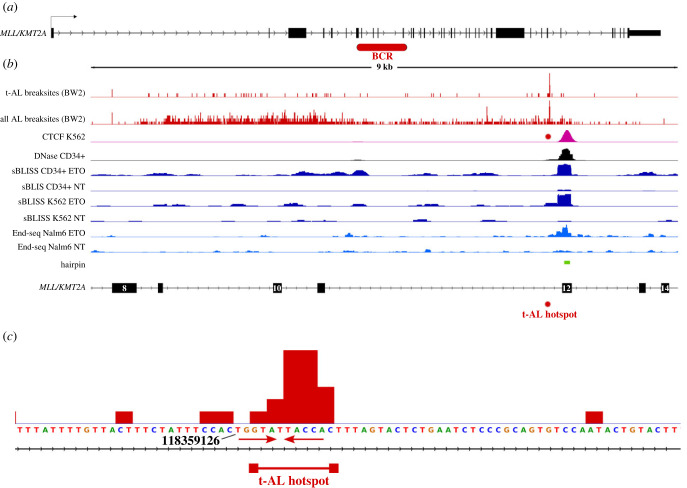
Genomic and epigenetic landscape of the *MLL* BCR. (*a*) Intron-exon arrangement of the *MLL*/*KMT2A* gene, with the position of the major AL-associated BCR highlighted in red. (*b*) Exon 8 to 14 region of the *MLL / KMT2A* gene. t-AL break sites, mapped der(11) *MLL* translocation break sites; all AL break sites, mapped der(11) *MLL* translocation break sites from t-AL and de novo AL combined. Break site positions are from [2,20–25]. Genomic data (hg19) are from the following sources. CTCF K562, CTCF RAD21 and DNase CD34^+^, ENCODE [26]; End-seq ETO, Nalm-6 End-seq in the presence of etoposide, GEO GSE99194 [27]; End-seq NT, Nalm-6 End-seq not treated, GEO GSE99194 [27]; sBLISS data GEO121742 [28], hairpin [29]. (*c*) Enlargement of the t-AL hotspot and flanking region highlighting an inverted repeat coinciding with the hotspot. hg19 coordinate of the 1st nucleotide of the t-AL hotspot is indicated. For (*b*) and (*c*) break site counts were binned (binning = 2 nt) and plotted in histogram form.

*MLL* translocation junction break sites (*der-11*) found in individual acute leukaemia (AL) cases fall within an 8.3 kb breakpoint cluster region (BCR), spanning intron 8 to exon 12 of the *MLL* gene (figure 2*a*). In *de novo* AML cases (i.e. not associated with prior exposure to clastogenic anti-cancer drugs), *MLL* breakpoints cluster in a broad area towards the centromeric (5′) half of the BCR. However, in t-AL and neonatal acute leukaemias the *MLL* junction sequences are concentrated in the telomeric (3′) 1 kb of the BCR (figure 2*b*), suggesting that an additional or alternative mechanism is involved in their formation [2,20,30,31]. Furthermore, nucleotide resolution mapping of translocation break sites from t-AL patients has identified a hot-spot close to the 3′-end of intron 11 (figure 2*b*,*c*). This hotspot almost overlaps with (within approx. 300 bp from the centre of) a DNase I hypersensitive region found in exon 12 that is occupied by CTCF [19,27,32]. Notably, CTCF interacts with TOP2B and in genome-wide studies a significant number of CTCF sites are also occupied by TOP2B and are vulnerable to TOP2 poison-induced DNA cleavage, leading to the conclusion that such CTCF/TOP2 occupied regions could drive genome fragility [27,28,33]. Indeed, these whole-genome studies have demonstrated specific DNA cleavage at the exon 12 DNase hypersensitive/CTCF site in cells exposed to etoposide (figure 2*b*, electronic supplementary material, figure S1). This is broadly consistent with older publications reporting specific TOP2 poison-induced cleavage in cell line systems at, or at least near this t-AL translocation hot-spot region [32,34–38]. These studies employed southern blotting or PCR-based methods to map sites of TOP2 poison-mediated cleavage within the 8.3 Kb BCR. Some of these studies have been interpreted as evidence for the type of translocation mechanism described above, that is, erroneous repair of DSBs generated directly by TOP2. However, the doses of TOP2 poison employed and the prolonged time after addition of the agent in these earlier studies (typically 4–16 h) suggests that events secondary to immediate TOP2 poison-induced DSB formation may be involved, and indeed there is evidence that TOP2 poison induced DNA cleavage in this region could be related to early apoptotic events [35–37,39,40]. Notably, apoptotic fragmentation of genomic DNA starts with the cleavage of high mw chromosomal loops, a process that may involve TOP2 as well as apoptotic nucleases including Endo G [41–45]. For this early apoptotic cleavage to contribute to leukaemogenesis, it would be necessary for affected cells to sometimes recover from early apoptotic events and re-enter the cell cycle, a process that would require extensive DNA repair with the opportunity for chromosomal rearrangement. Perhaps surprisingly, there is a growing body of evidence that this can be the case at least in cell culture systems, and this recovery from early apoptotic events has been termed anastasis [46,47].

Thus, although the mechanism(s) leading to these *MLL* recombination events are not clearly understood, candidate processes include (i) rearrangement resulting from miss-repair of TOP2 poison-induced TOP2-mediated DNA DSBs in haematopoietic progenitor cells and (ii) recovery from early apoptotic events that result in cleavage within the *MLL* BCR and subsequent repair and recombination. In addition, DNA secondary structure effects have also been suggested to contribute to the distribution of breakpoints within the *MLL* BCR. Furthermore, these mechanisms are not necessarily mutually exclusive.

An opportunity now exists to better understand the molecular events leading to TOP2 poison-related *MLL* chromosome translocations by comparing recently published genome-wide mapping of etoposide-induced TOP2 CCs and DNA DSBs with the nucleotide resolution pattern of der(11) *MLL* translocation breakpoints from t-AL patients in combination with previous data concerning sites of early apoptotic cleavage in the *MLL* BCR.

## 2. A hotspot for t-AL derived MLL translocation break sites maps close to but separate from the exon 12 DNase hypersensitive / CTCF site

It has been known for some time that *MLL* translocation breakpoints associated with prior exposure to TOP2 poisons cluster at the telomeric (3′) end of the BCR (figure 2*b*) [2,31]. As more examples have been identified and mapped at nucleotide resolution, an 11 bp hotspot for t-AML break sites has emerged close to the *MLL* intron 11/exon 12 boundary [2,21,22] (see figure 2*b*,*c*). At the same time a DNase hypersensitive site was reported in this region, and restriction enzyme/Southern blotting analysis [32] revealed *in situ* DNA cleavage induced by etoposide and other TOP2 poisons in the same region in cultured cells. We and others have subsequently shown that this exon 12 DNase hypersensitive region corresponds to a strong CTCF binding site [19,27,48]. Data from recent whole genome studies using the related methods of End-seq [27] and BLISS [28,49] to map genomic sites of DNA DSB formation have allowed high resolution mapping of etoposide-induced DNA breaks in the *MLL* locus. Inspection of these data confirms efficient cleavage within exon 12, closely associated with site of CTCF/RAD21 binding and DNase hypersensitivity (figure 2*b*) and overlapping a previously reported TOP2 cleavage consensus sequence [34]. This finding is consistent with the established protein interaction between TOP2B and CTCF and observed overlap between peaks of TOP2B and CTCF chromatin occupancy in ChIP-seq experiments [27,33,50]. Putting these observations together, it has been concluded that such TOP2/CTCF binding sites are regions of genome fragility, especially in cells exposed to TOP2 poisons, and that this can directly contribute to clinically relevant chromosome translocations such as those observed in t-AML. However, comparing the precise location of the t-AL break site hotspot with the position of CTCF/RAD21/DNase hypersensitivity /End-seq peaks revealed that the hotspot is considerably offset. Comparing the centre of the CTCF/RAD21 peak and CTCF binding motif with the centre of the break site hotspot, the offset is 266 bp, with the hotspot centromeric (5′), while the spacing is 230 bp from the major End-seq and BLISS peaks to the hotspot (electronic supplementary material, figure S1). While some degree of DNA resection might be involved in the accidental joining of heterologous chromosome fragments leading to chromosome translocation, this does not seem a likely mechanism to account for the offset, especially as the break sites are so tightly clustered in the hotspot. This suggests that an alternative or additional mechanism is involved that accounts for the clustering of the break sites in the hotspot.

## 3. DNA secondary structure

The tendency to form DNA secondary structures has been suggested as a possible factor in AL break site selection [29,51], and for TOP2-mediated DNA cleavage and genomic instability [52,53]. Intriguingly a putative hairpin structure maps to exon 12 coinciding with CTCF/RAD21/DNase HS/End-seq peak described above (figure 2*b*) [29]. While this structure is located downstream of the t-AL hotspot (see above), we noticed that the 11 bp sequence directly underlying the hotspot (TGGTATTACCA) is itself a short palindrome (figure 2*c*), although the significance of this is not clear. Next, we determined the relationship between the *MLL* BCR, and specifically the t-AL hotspot and tendency to form R-loops and G-quadruplex structures. As shown in electronic supplementary material, figure S2, examination of published DRIPc R-loop mapping derived from K562 cells [54] revealed extensive R-loop formation within the BCR region. This mostly mapped to the 5′ third of the BCR, although it does partially overlap the most common region for de-novo AL translocation break sites. By contrast, G4-seq data [55] highlighted the propensity for template strand quadruplex formation at just one site in the BCR immediately upstream of the t-AL hotspot (electronic supplementary material, figure S2). This G4-seq peak contains a likely candidate quadruplex forming sequence centred on position 118 359 010 corresponding to a nucleotide position just 120 nucleotides upstream of the centre of the t-AL hotspot.

## 4. Preferential TOP2 cleavage sites

**Figure 3.**
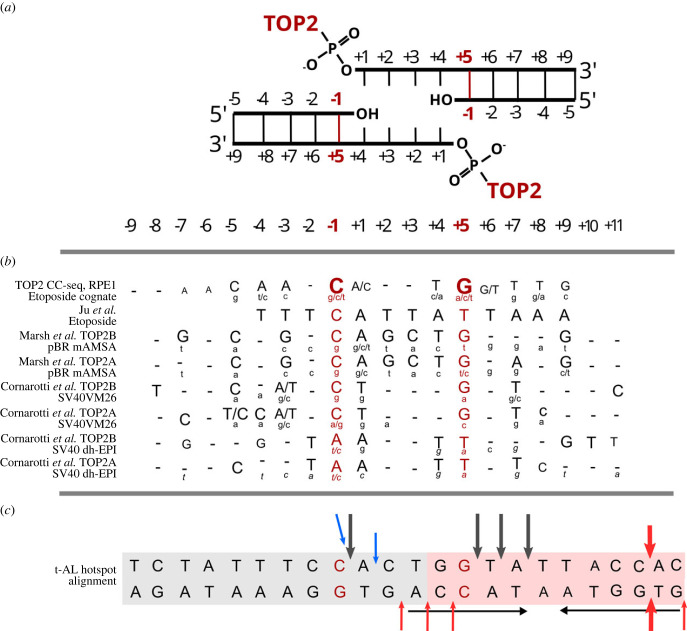
Comparison of TOP2 DNA cleavage site preferences and the t-AL hotspot sequence. (*a*) Illustration of a TOP2CC highlighting the 4 bp DNA overhang. Numbering is aligned with the numbering and sequences in part (*b*). Cleavage occurs between base −1 and +1 on both the Watson and Crick strands. (*b*) Base composition preferences or TOP2 DNA cleavage. Cleavage sites and base composition preferences are derived from the following sources: TOP2 CC-seq (Gittens *et al.*) [56], Ju *et al.* [57], Marsh *et al.* [58], Cornarotti *et al.* [59]. −1 and +5 positions are highlighted in red for clarity. Upper case lettering indicates over-representation at a given position, bold for more prominent overrepresentation; lower case indicates under-representation or absence at the given position. Dash represents no identified over/under representation. For TOP2 CC-seq the base preferences are derived from the average base composition of cognate TOP2 CC sites genome-wide in etoposide treated RPE1 cells [56]. (*c*) Partial alignment of the t-AL hotspot (shaded pink) and its adjacent 5′ flanking sequence (shaded grey) with cleavage site base composition preferences in (*b*), including the position of the inverted repeat (horizontal arrows). Black vertical arrows, *in vitro* etoposide-induced cleavage sites identified in [22]; blue arrows, in cell etoposide-induced cleavage sites identified in [36]; red arrows, apoptosis-associated DNA breaks identified in [36].

TOP2 makes a staggered double-stranded DNA break with a 4 bp 5′-overhang that remains attached to the enzyme protomer via a phosphotyrosyl linkage until re-ligation (see figures 1*b* and 3*a*). These cleavage complexes are stabilized in the presence of TOP2 poisons, allowing analysis of sites of DNA cleavage. Neither TOP2A nor TOP2B display strong DNA cleavage site preference *in vitro*. However, mapping of cleavage patterns in plasmid or viral DNAs [58,59] and more recently analysis of genome-wide TOP2 CC formation in RPE-1 cells (CC-seq) [56] have revealed some consensus features, most notably a dyad symmetrical preference for cytosine at position −1 (the base immediately 5′ of the cleaved phosphodiester bond) on both strands in the presence of etoposide, VM26 or mAMSA and adenine at the same positions in the presence of dh-EPI (figure 3*b*). Thus, the question arises as to whether the 11-bp hotspot sequence or sequences immediately surrounding it constitutes a preferable TOP2 cleavage site or matches the TOP2 cleavage preferences described above. One of the first *MLL* t-AL break sites that was mapped in this region at base-pair resolution was reported by Whitmarsh *et al.* [22]. In this study, the authors demonstrate using *in vitro* cleavage assays that this region is a target for TOP2A-induced cleavage associated with etoposide or its catechol and quinone metabolites (figure 3*c*). Notably, only one of the four *in vitro* cleavage sites from this study [22] that map within or very close to the t-AL hotspot matches the preferred cleavage base composition described above (figure 3*b,c*). However, other sites further from the hotspot were also cleaved with similar efficiency. While this evidence demonstrates that TOP2-mediated cleavage can occur within and very near to the 11 bp hotspot, *in vitro* cleavage site mapping and whole genome CC-seq and DNA break mapping (figure 3*b,c*, electronic supplementary material, figure S1) does not support the hypothesis that the hotspot is an especially high-efficiency TOP2 cleavage site by virtue of its DNA sequence to an extent that could explain the focused distribution of *MLL* t-AL translocation break sites.

## 5. Apoptotic mechanisms

Early apoptotic cleavage has also been proposed as a factor in intron 11/exon 12 cleavage observed in cells and the clustering of t-AL *MLL* break sites. Data underlying this hypothesis includes relatively low-resolution Southern blot analysis to detect *MLL* gene cleavage [31,34,37]. Using this approach similar intron 11/exon12 cleavage patterns were observed when cells were treated with TOP2 poisons, non-TOP2 targeting DNA damaging agents, or indeed the pro-apoptotic anti-CD95 antibody [37,39]. The sizes of the resulting restriction fragments detected by Southern blotting appears to localize the primary site of cleavage (at 1.5 kb from a BamHI site in intron 12) at the exon 12 DNase/CTCF/End-seq/sBLISS site described above. However, base-pair resolution mapping of etoposide-induced direct cleavage and apoptotic scission in human P6 lymphoblastoid cells obtained using extension ligation-mediated PCR yielded a more complex pattern [36]. While the strongest apoptotic cleavage was observed within exon 12, a secondary cluster of direct etoposide and apoptotic cleavages were mapped further 5′, overlapping with the t-AL breakpoint hotspot. Notably, this secondary cluster contained an etoposide-induced direct cleavage site at the same position as one of the in sites determined *in vitro* by Whitmarsh *et al.* [22] and several sites of apoptosis-related cleavage within the 11 bp hotspot including an apparent blunt-ended cleavage close to the 3′ end of the hotspot (see figure 3*c*).

## 6. TOP2A or TOP2B

Using break-apart DNA FISH probes we previously showed that TOP2B is required for efficient etoposide-mediated induction of breaks in the *MLL* locus in Nalm6 pre-B cell leukaemia cells [19]. However, it has also recently been reported that both TOP2B and TOP2A can contribute to etoposide-induced *MLL* locus breakage, but that the contribution of each isoform depends on their relative abundance [28]. We have previously shown that there are similar numbers of TOP2A and TOP2B protein molecules in cultured leukaemic cell lines [60]; however, the question arises as to the relative expression levels of TOP2A and TOP2B *in vivo* in haematopoietic stem cells and lymphoid and myeloid progenitors in which leukaemias arise. Examination of RNA-seq datasets (electronic supplementary material, figure S3, BLUEPRINT Consortium) [61] revealed that the expression of TOP2B RNA is over ten times higher than that of TOP2A in human haematopoietic stem cells and in multipotent, lymphoid and myeloid progenitors, consistent with the largely quiescent status of HSC cells [62]. This leads to the conclusion that TOP2B is likely to be the major contributor to TOP2 poison induced *MLL* cleavage in haematopoietic progenitors *in vivo*.

## 7. Conclusion

**Figure 4.**
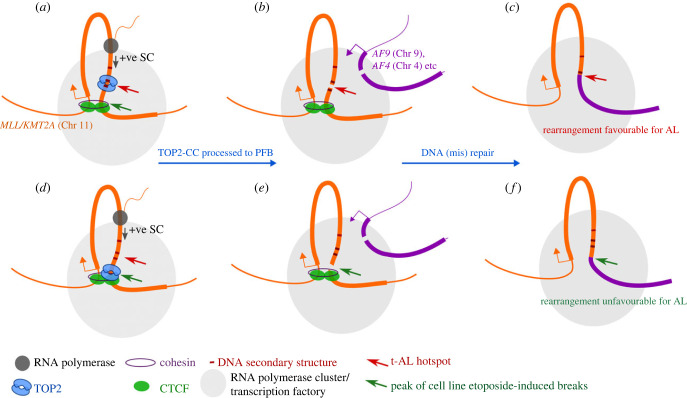
Model for reciprocal *MLL* chromosome translocation upon exposure to TOP2 poisons. Chr11 segment (orange line) containing the *MLL/KMT2A* gene (thick section) depicted with CTCF/cohesin-mediated looping between the exon 12 CTCF site and an upstream region (CTCF depicted as green ovals, and cohesin as a purple ring). Elongating RNA pol II (dark grey circle) progressing towards the topological constraint of the loop base generates positive superhelical tension upstream of the exon 12 CTCF site that requires topoisomerase activity (blue) for resolution (*a*). In the presence of a TOP2 poison, normally transient TOP2-DNA complexes associated with strand passage activity are stabilized, and subsequently processed into protein-free DSBs (PFB, red arrow) (*b*), which in turn can lead to chromosome rearrangements via erroneous DNA repair (*c*). We suggest that the observed clustering of t-AL translocation break sites in the 11-bp hotspot (bounded by dark bars) results from a combination of DNA sequence permissive to TOP2 cleavage, DNA secondary structure features and factors such as nucleosome positioning, along with the constraint of generating a rearrangement favourable for AL development (*a–c*). In this model, balanced chromosome translocation is facilitated by the proximity of a translocation partner gene undergoing transcription in the same RNA polymerase cluster/transcription factory (large light grey circle) as *MLL/KMT2A.* In parallel, etoposide can also lead to DSBs associated with TOP2B at the exon 12 CTCF site (*d*), accounting for the peak of etoposide-induced breaks detected by End-seq and BLISS associated with this region (green arrow) (*e*). However, t-AL associated *MLL/KMT2A* translocations involving break sites at this location (*f*) are not frequently observed, presumably as they are less likely to be favourable for AL development.

It is intriguing that while etoposide and indeed other cytotoxic treatments can efficiently induce DNA cleavage in the *MLL* BCR in cell line-based studies, this cleavage is focused primarily on the CTCF/RAD21/DNase HS region in exon 12 rather than the nearby t-AL hotspot in intron 11 that represents multiple patient translocation break sites. Thus, although the capacity for DNA cleavage can be demonstrated in the 11 bp hotspot (figure 3) [22,36], the distribution of *MLL* break sites observed in t-AL cases cannot be explained simply by the most frequent sites of DSB induction by TOP2 poisons. Several possibilities suggest themselves to explain this positional discrepancy. Firstly, since *MLL* translocation break sites are derived from t-AL cases, the resulting der(11) encoded *MLL* fusion genes must generate mRNAs that are translated into proteins that can deregulate and transform blood cell progenitors. Few translocations have been mapped in exon 12 in either t-AL or de novo cases, suggesting that translocations involving exon 12 are less favourable in this respect. This extra constraint would favour DSBs upstream of exon 12. Notably, End-seq carried out in Nalm-6 human lymphoblastoid cells [27] and sBLISS data from K562 cells [28] both show the presence of etoposide-induced DNA breaks upstream of the peak cleavage signal, extending towards and overlapping the t-AL hotspot (figure 2; electronic supplementary material, figure S1). In addition, genome-wide CC-seq data [56], which maps sites of etoposide stabilized TOP2 covalent complexes at base pair resolution, provides evidence for TOP2 CC stabilization at the site of the t-AL hotspot as well as coincident with the CTCF/RAD21/DNase HS region (electronic supplementary material, figure S1). Thus, at least part of the explanation for the appearance of a tight hotspot for t-AL associated translocations could be the combination of a favourable site for functional fusion gene generation and TOP2 poison-induced TOP2 CC stabilization and subsequent processing to DSBs. In addition, we noticed that a prominent Pyridostatin stabilized G-quadruplex [55] maps just upstream (120 nucleotides) of the t-AL hotspot, in an orientation such that the G-rich sequence would be present in the template strand. Although the significance of this feature is not clear it has recently been reported that TOP2 contributes to the observed genomic instability associated with DNA secondary structure features [52]. In addition, the presence of a prominent CTCF peak in *MLL* exon 12 raises the possibility that CTCF-mediated chromatin looping may play a role in break site specification at the 3′ end of intron 11. Indeed, it has been noted [28] that from ChIA-PET data, the exon 12 CTCF site is associated with a loop anchor region that establishes contacts with upstream regions (figure 4). Furthermore, in the configuration of the *MLL* BCR (i.e. transcription towards the CTCF site, coming from inside the loop), aggregate data would predict a prominent enrichment of TOP2-induced DSBs at a mean distance of 45 nt 5′ of the CTCF motif [28]. This is also consistent with TOP2-induced DSBs enriched around the strongly positioned nucleosomes of CTCF sites reported for mouse B lymphocytes [27]. Although the 45 nt spacing mentioned above, between CTCF motif and maximum DSB enrichment is less than the observed spacing between this motif and the t-AL hotspot (266 nt), it should be noted that the observed spacing is close to that occupied by two nucleosomes positioned as a result of CTCF binding. Furthermore, topoisomerases including TOP2 are required to modulate DNA supercoiling effects ahead of and behind an elongating RNA polymerase. The presence of a CTCF-associated loop-anchor region in exon 12 of *MLL* may impede dissipation of positive supercoiling tension as the polymerase approaches exon 12, requiring localized TOP2 activity (figure 4*a*). This localized activity could then be converted to TOP2-CCs and ultimately protein-free DSBs in the presence of TOP2 poison-containing anti-cancer regimens (figure 4*b*). This transcription-linked mechanism is consistent with the previously reported observation that *MLL* and its frequent t-AL translocation partners *AF4* and *AF9* can be transcribed in very close proximity, potentially in shared RNA polymerase clusters/transcription factories, facilitating chromosome translocation when DNA breaks are present in both partner genes [19] (figure 4*c*). In parallel, the association of TOP2B with CTCF explains the exon 12 etoposide-mediated DSB signal observed in End-seq and sBLISS studies (figure 2*b*; figure 4*d–f*). However, these considerations alone do not explain the concentration of *MLL* break sites in the t-AL hotpot. Other factors that potentially lead to the observed clustering include DNA cleavage site preferences, G-quadruplexes flanking the hotspot or the underlying short palindrome affecting TOP2 cleavage and CC formation, or the requirement to generate a chromosome translocation that is favourable for clonal expansion and leukaemia initiation. Furthermore, although it is tempting to assume that the clustering of t-AL break sites is related to sites of efficient or directed TOP2-mediated cleavage in this region, the low frequency of t-AL cases may also point to the importance of minor cleavage sites in their aetiology.

It has also been proposed that *MLL* break site selection results from early apoptotic events, rather than immediate CC formation and their processing to DSBs as discussed above. Early apoptosis is characterized by the formation of 50–100 kb chromatin fragments that may be derived from chromatin loops, and appear to be at least partially dependent on TOP2 [43–45,63], although notably, TOP2 is also reported to interact with CAD nuclease [64]. High molecular weight early apoptotic cleavage is also consistent with the earlier Southern blot-based data highlighting efficient apoptosis-associated cleavage mapping to the CTCF/RAD21/DNase HS region. Furthermore, in one study, nucleotide resolution mapping of apoptotic cleavage sites in the Intron 11-Exon 12 region of *MLL* [36] revealed a secondary cluster of apoptotic cleavage overlapping with the t-AL hotspot. For early apoptotic cleavage to account for *MLL* translocations observed in t-AL, there must be a possibility for cells to recover from these early events and continue to proliferate. Recently, evidence has been found that cells can sometimes recover after initiation of apoptosis, in a process known as anastasis [46]. Not surprisingly considering the prevalence of apoptotic nucleases, this phenomenon is associated with genomic instability and oncogenic transformation [65]. In addition, CAD-mediated reversible DNA cleavage in the vicinity of CTCF sites has been reported to have a role in maintaining cell cycle checkpoint activation following DNA damage [66].

To summarize, Southern blot data which probably reflects early apoptotic cleavage after prolonged TOP2 poison exposure and next-generation break mapping techniques (End-seq and sBLISS) after short etoposide exposure both show efficient etoposide-induced DNA cleavage in *MLL* exon 12. Although it is tempting to explain the existence of the t-AL translocation hotspot by this DNA cleavage pattern, closer examination of the genomic positions of these two features is not consistent with this conclusion without considering further constraints. While no feature that we considered can explain the t-AL hotspot on its own, we suggest that the observed t-AL break site distribution results from a combination of TOP2B-mediated strand breaks associated with the 5′-side of the exon 12 CTCF site (5′ of the peak of DNA cleavage) and the requirement for any *MLL* recombination products to generate a functional fusion protein capable of transforming haematopoietic progenitors. The factor/s that favour the precise position of the 11 bp t-AL hotspot remain unclear beyond the conclusion that the major site of DNA cleavage in exon 12 is unfavourable for functional fusion protein formation. This analysis also does not determine whether the DNA breaks involved in the patient *MLL* rearrangements originate directly from TOP2 poisoning and conversion of TOP2–DNA complexes to protein-free breaks, or from early apoptotic cleavage events, but these processes are not mutually exclusive, and the data considered here supports both possibilities. This means that the jury is still out as to the detailed mechanism for site selection of the t-AL fusion junction in *MLL* translocations.

## Data Availability

This article includes re-analysis of genomic data published elsewhere, and deposited by the originators in the Gene Expression Omnibus (https://www.ncbi.nlm.nih.gov/geo/). Accession codes for each data set used are given at the appropriate point in the manuscript. Sources of translocation break site positions are also fully described in the text. The data are provided in electronic supplementary material [67].
